# Carotid artery atherosclerosis, MRI-defined structural brain abnormalities, and cognitive performance in elderly American Indians: The Strong Heart Study

**DOI:** 10.3389/fepid.2025.1659856

**Published:** 2025-10-07

**Authors:** Tauqeer Ali, Dedra Buchwald, Dean Shibata, Mary J. Roman, Steven Verney, Barbara V. Howard, Jason Umans, Shelley Cole, Cynthia West, Ying Zhang, Jessica Reese, Dorothy A. Rhoades, Marcia O'Leary, W. T. Longstreth, Amanda Fretts, Astrid Suchy-Dicey

**Affiliations:** ^1^ Department of Biostatistics and Epidemiology, Center for American Indian Health Research, Hudson College of Public Health, University of Oklahoma Health Sciences Center, Oklahoma, OK, United States; ^2^ Neurosciences Institute, Department of Neurological Surgery, UW Medicine, University of Washington, Seattle, WA, United States; ^3^ Department of Radiology, University of Washington, Seattle, WA, United States; ^4^ Division of Cardiology, Department of Medicine, Weill Cornell Medical College, New York, NY, United States; ^5^ Department of Psychology, University of New Mexico, Albuquerque, NM, United States; ^6^ MedStar Health Research Institute, Hyattsville, MD, United States; ^7^ Texas Biomedical Research Institute, San Antonio, TX, United States; ^8^ Department of Medicine, College of Medicine, University of Oklahoma Health Sciences Center, Oklahoma, OK, United States; ^9^ Missouri Breaks Industries Research Inc., Eagle Butte, SD, United States; ^10^ Department of Neurology, University of Washington, Seattle, WA, United States; ^11^ Department of Epidemiology, School of Public Health, University of Washington, Seattle, WA, United States; ^12^ Slone Epidemiology Center, Boston University School of Medicine, Boston, MA, United States

**Keywords:** carotid artery atherosclerosis, MRI-defined structural brain abnormalities, sulcal widening, cognitive performance, American Indian population

## Abstract

**Background and objective:**

American Indian populations face disproportionately high rates of atherosclerotic cardiovascular disease (CVD), yet the potential consequences of mid-life carotid atherosclerosis on brain health and cognition later in life remain poorly understood. This study addresses a critical knowledge gap by evaluating whether subclinical carotid atherosclerosis in midlife is associated with later-life structural brain abnormalities and cognitive performance in a large cohort of American Indian adults from the Strong Heart Study. This is the first investigation to explore these associations in this underserved and understudied population, using longitudinal data with vascular, neuroimaging, and cognitive measures.

**Methods:**

A total of 783 participants (mean age 59.9 years) underwent carotid ultrasonography between 1998 and 1999 to assess intima-media thickness and plaque. Between 2010 and 2013, participants received brain magnetic resonance imaging to assess infarcts, hemorrhages, white matter lesions, and brain atrophy. Cognitive function was also evaluated during this period. Multivariable regression models adjusted for sociodemographic, behavioral, and clinical CVD risk factors were used to assess associations.

**Results:**

Greater intima-media thickness was associated with more severe sulcal widening, and presence and extent of plaque were associated with poorer verbal fluency; both findings remained significant after adjustment for sociodemographic, behavioral, and clinical risk factors. No significant associations were observed between carotid measures and the presence of infarcts, hemorrhages, or white matter lesions.

**Conclusion:**

These findings suggest that subclinical carotid atherosclerosis in midlife may contribute to later-life brain atrophy and cognitive vulnerability, particularly in verbal fluency, among American Indians.

## 1 Introduction

American Indians exhibit the highest prevalence of carotid artery stenosis across age groups and sexes (1), likely due to the high burden of cardiovascular disease (CVD) risk factors in this population (2, 3). These risk factors, particularly type 2 diabetes, hypertension, obesity, and smoking, are more prevalent and often occur at younger ages or with greater severity among American Indians than in other U.S. racial and ethnic groups (4, 5). While cholesterol levels may not be elevated on average in this population, both LDL and non-HDL cholesterol have been recognized as independent predictors of coronary heart disease, especially among individuals with diabetes (6). As a result, American Indians experience the highest stroke prevalence compared to the broader U.S. population (3).

In addition to the elevated burden of overt stroke, emerging evidence suggests that American Indian adults may also bear a substantial burden of subclinical neurovascular and neurodegenerative brain changes. Studies have reported a high prevalence of MRI-defined structural brain abnormalities in older American Indians (7–9). These structural brain changes are well-established markers of cerebral small vessel disease and neurodegeneration and are associated with poorer cognitive outcomes. In a recent population-based study, cognitive impairment was identified in over half of American Indian participants aged 70–95 years, with 35 percent meeting criteria for mild cognitive impairment (MCI) and 10 percent for dementia (10). The MCI rate exceeds the range reported in non-Hispanic White populations (12–28 percent) (10). Supporting this pattern, a separate multiethnic analysis of older U.S. adults reported that American Indian and Alaska Native individuals had a significantly higher risk of developing MCI compared to non-Hispanic White people, with a subhazard ratio of 1.73 (*p* < 0.05) after adjustment for demographic and health-related factors (11). Together, these findings highlight a pressing need to better understand how the high burden of vascular risk factors among American Indians may contribute to structural brain abnormalities and poorer cognitive outcomes later in life.

Despite the recognized need to better understand vascular contributions to brain aging, the role of subclinical carotid atherosclerosis in midlife as a determinant of later-life structural brain abnormalities and cognitive performance in American Indians remains poorly understood. To our knowledge, no prior study has evaluated these associations in this population using longitudinal data. Improved insight into these relationships could help identify modifiable early-life vascular risk factors contributing to disparities in cognitive and brain aging outcomes.

In this study, we used data from the Strong Heart Study (SHS) to evaluate whether subclinical carotid atherosclerosis in midlife, measured via carotid ultrasound in 1998–1999, was associated with structural brain abnormalities and cognitive performance assessed approximately 13 years later, between 2010 and 2013. To our knowledge, this is the first longitudinal investigation of these associations in a large population-based sample of older American Indian adults.

## 2 Materials and methods

### 2.1 Study population

The SHS is a population-based, ongoing longitudinal study of prevalent and incident cardiovascular disease and its risk factors originally involving 4549 American Indian tribal members living in 13 communities in the Northern Plains, Southern Plains, and Southwest regions of the US (12). Participants were between the ages of 45 and 74 years when they were enrolled in the first examination between 1989 and 1991. The cohort was re-examined approximately every 5 years through 1999.

### 2.2 Carotid ultrasound measurements

Carotid ultrasound was performed on all surviving and able SHS participants during the third examination (1998–1999) using a standardized protocol (13).

The following 3 vascular measurements were used for these analyses: The presence of atherosclerotic plaque in the carotid artery, which was defined as focal carotid arterial wall thickening >50% compared to the thickness of the surrounding wall (14). A carotid plaque score was calculated by counting the number of segments containing plaque, combining the left and right common carotid arteries, carotid bulbs, and external and internal carotid arteries. Carotid plaque scores ranged from 0 (no plaque in any segment of either artery) to 8 affected segments. The presence of plaque in the external carotid artery was included in the calculation of the plaque score, as previous research has indicated that external carotid plaques are associated with intracranial stenosis (15). Intima-media thickness of the far wall of the common carotid artery was measured at end-diastole on several cycles and then averaged (16). Wall thickness was not measured at the level of a plaque. Left and right wall thicknesses were averaged and the mean thickness of the 2 (in mm) was calculated.

### 2.3 Brain MRI measurements

Between 2010 and 2013, 1,033 surviving participants aged 64 and older were enrolled in the “Cerebrovascular Disease and its Consequences in American Indians (CDCAI) study” (17), an SHS ancillary study designed to assess vascular brain injury. Brain MRI and cognitive function were assessed. The average time interval between the carotid ultrasound examination (conducted in 1998–1999) and the brain MRI assessment was 13.2 years.

The protocol for acquiring and processing MRI scans has been described previously (18). A study neuroradiologist trained in the CDCAI imaging protocol and blinded to participant data read and graded all scans.

The following brain MRI measures assessed in CDCAI were used in this analysis:

Brain infarcts were defined as lesions 3 mm or larger (including both lacunes and larger cortical infarcts) with characteristic shape and signal intensity. Infarcts were required to have hyperintensity to gray matter on both T2-weighted images and FLAIR images to contrast with perivascular spaces, which have characteristic location and shape and demonstrate cerebrospinal fluid (CSF) intensity on all sequences, but hypointensity on T1-weighted images, to distinguish them from focal white matter hyperintensities (WMH) (19). Infarct lesions were defined as lacunar if they were between 3 and 20 mm in maximum dimension and located within the caudate, lenticular nucleus, internal capsule, thalamus, brainstem, cerebellar white matter, centrum semiovale, or corona radiata (7). Non-lacunar infarcts included cortical infarcts and large subcortical infarcts.

Brain hemorrhages were defined as lesions following blood product signal intensities on the T1-and T2-weighted images and hypointense “blooming” on T2* susceptibility-weighted images, which are particularly sensitive to even small amounts of hemosiderin (7). Both microhemorrhages and larger hemorrhages were recorded.

Severity of WMH, sulcal widening, and ventricle enlargement were graded using a semi-quantitative 10-point scale based on previously-validated image standards using FLAIR images for WMH and T1-weighted images for sulci and ventricles grading (20). To determine the best visual fit for severity, a neuroradiologist matched participants' scan images to a set of similar standard templates used by the Atherosclerosis Risk in Communities study and the Cardiovascular Health Study. Grades ranged from 0 (absence of disease) to grade 9 (most severe) (21). A grade of 3 or higher was considered abnormal.

### 2.4 Cognitive testing

Cognitive testing was conducted during the same visit as the brain MRI, approximately 13.2 years after the carotid ultrasound assessments. The following cognitive testing measures assessed in CDCAI were used in this analysis:

Four standard cognitive tests were administered: (1) The Modified Mini Mental State Examination (3MSE) is a widely used screening tool for general cognitive functioning (22). (2) Weschler Adult Intelligence Scale (WAIS) 4th edition coding subtest, measuring visuospatial processing speed and working memory (23). (3) Controlled Oral Word Association Test (COWAT) using the letters F, A, and S to evaluate phonemic verbal fluency and executive functioning (24). (4) California Verbal Learning Test (CVLT) version II short form, comprising several indices of immediate and delayed verbal learning and memory (25).

### 2.5 Baseline covariates from the SHS third examination cycle (1998–1999)

Covariates were derived from the SHS Third Examination Cycle (1998–1999), considered the baseline for this analysis, as carotid ultrasounds were performed then.

Participants completed questionnaires, self-reporting their sex (categories included female and male), years of formal education (assessed in SHS first examination cycle), and smoking status (ever vs. never smoked). Ever smoke was defined as “smoked at least 100 cigarettes in entire life”; participants also entered the age they started smoking and reported if they do not smoke currently. Participants who never smoked was defined as “having not smoked more than 100 cigarettes in entire life or never smoked regularly.” Alcohol use was categorized as ever vs. never. The former was defined as “having more than 12 drinks in entire life.” The latter was defined as “never having consumed alcoholic beverages.”

History of CVD (coronary heart disease, heart failure, atrial fibrillation) was determined based on adjudication procedures using systematic review of medical records.

Anthropometric measurements included in this analysis include weight and height. Body mass index (BMI) was calculated as body weight divided by height squared (kg/m^2^).

Three consecutive measurements of blood pressure were performed. The mean of the last 2 measurements was used to estimate blood pressure. Hypertension was defined as self-reported current antihypertensive therapy or by JNC 7 criteria: systolic blood pressure (SBP) ≥ 140 mmHg or diastolic blood pressure (DBP) ≥ 90 mmHg.

Participants fasted for at least 12-h overnight. A 75-g oral glucose tolerance test was performed on all participants except for participants with diabetes treated with insulin or oral hypoglycemic agents or those with fasting glucose ≥225 mg/dl as determined by an Accu-Check II (Baxter Healthcare Corporation, Deerfield, IL).

ADA 2003 Standard of Care guidelines were used to define diabetes mellitus (i.e., fasting glucose ≥7.0 mmol/L or 126 mg/dl, post-oral glucose challenge glucose measurement of ≥11.1 mmol/L or 200 mg/dl, or the use of oral hypoglycemic medications or insulin to treat diabetes).

LDL-C was derived using the Friedewald equation (26); it was directly measured in participants with triglyceride values of ≥400 mg/dl.

All participants gave written informed consent. Tribal authorities, the Indian Health Service, and Institutional Review Boards for participating communities and partner institutions approved study protocols for the SHS and CDCAI.

### 2.6 Statistical analysis

Inclusion in these analyses was determined based on having participated in both the baseline SHS and follow-up CDCAI examinations. For this analysis, 215 participants were removed because one community withdrew consent; 29 participants were excluded based on incomplete or inadequate brain MRI scans; 3 participants were excluded due to incomplete cognitive testing; 3 participants were removed during the adjudication process due to a history of prior stroke. Stroke is a known independent cause of and contributor to cognitive impairment. Thus, the final analytic sample consisted of 783 participants.

Multivariable regression analyses were used to determine the associations of 3 measures of carotid atherosclerosis measures (CIMT, plaque, and plaque score) with brain MRI markers (infarct, hemorrhage, WMH grade, sulcal widening grade, and ventricle enlargement grade) and with cognitive testing scores (3MSE score, WAIS coding score, COWAT score, and CVLT long delay free recall score). Separate analyses were performed to investigate each of the 3 measures of carotid atherosclerosis.

Logistic regression analyses were performed for dichotomous outcomes (infarcts and hemorrhages) and results were given as OR with 95% confidence interval (95% CI). Linear regression analyses were used for continuous outcomes (cognitive test scores); Poisson regression analyses were used for the graded outcomes (WMH grade, sulcal grade, ventricle grade); results for linear and Poisson regression analyses were given as standardized beta coefficients (*β*) with 95% CI.

Potential confounders were selected based on prior knowledge of association with exposure and outcome variables and were used in regression models for adjustment. All regression models were progressively adjusted as follows: Model 1 was adjusted for age, sex, and study center. Model 2 was further adjusted for education (continuously as number of years), smoking status (ever vs. never), and alcohol consumption (ever vs. never). Model 3 was further adjusted for BMI, fasting glucose, SBP, and LDL. Model 4 was further adjusted for time interval between carotid ultrasound and acquisition of brain MRI and cognitive testing (time-between-examinations). Model 5 included only the participants who were CVD free at the third examination and was adjusted for the covariates included in Model 4. Model 6 for the cognitive testing scores was further adjusted for brain MRI markers. We did not include statin use as a covariate because LDL-C, a more direct and continuous measure of lipid-related cardiovascular risk, was already included in the models.

An alpha of ≤0.05 was used to test all hypotheses. Data analyses were conducted using Stata v.14 (StataCorp, 2014) or R version 3.3.1 (Team, 2016).

## 3 Results

Participant characteristics are presented in Table 1. At the third examination, participants in this analysis had a mean age of 59.9 years (SD: 5.8), 67.8% were female, mean years of completion of formal education was 12.3, body mass index was high (mean 31.6 kg/m^2^), 66.3% reported ever smoking, 82% reported ever consuming alcohol, 42.7% had hypertension, 48.7% had diabetes, mean LDL was 122.6 mg/dl, and 94% were free of CVD.

**Table 1:** Characteristics of participants.

Measures assessed at first SHS examination (1989–1991)	
Education (number of years): mean (SD)	12.3 (2.8)
Measures assessed at third SHS examination (1998–1999)	
Age (years): mean (SD)	59.9 (5.8)
Male participants: *n* (%)	252 (32.2)
Number of participants at the Study Centers: *n* (%)	
Southwest	91 (12.4)
Southern Plains	301 (41.1)
Northern Plains	340 (46.4)
Smoking status (ever): *n* (%)	477 (66.3)
Alcohol use status (ever): *n* (%)	588 (82.0)
BMI (kg/m2): mean (SD)	31.6 (5.9)
Systolic blood pressure (mmHg): mean (SD)	127.8 (16.8)
Diastolic blood pressure (mmHg): mean (SD)	75.7 (9.5)
Hypertension: *n* (%)	309 (42.7)
Diabetes: *n* (%)	232 (48.7)
Fasting glucose (mg/dl): mean (SD)	126.3 (51.8)
Serum LDL cholesterol (mg/dl): mean (SD)	122.6 (31.7)
Free of CVD at SHS 3rd cycle: *n* (%)	688 (94.0)
Time between SHS 3rd cycle and CDCAI exam (years): mean (SD)	13.2 (1.1)
Presence of plaques: *n* (%)	405 (57.0)
Plaque score: *n* (%)	
Score = 0	306 (43.1)
Score = 1	394 (55.5)
Score = 2	7 (1.0)
Score = 3	3 (0.4)
CIMT (mm): mean (SD)	0.7 (0.1)
Measures assessed at CDCAI examination (2010–2013)	
Infarcts (≥3 mm): *n* (%)	260 (33.3)
Hemorrhage: *n* (%)	45 (5.8)
White matter hyperintensity grade (≥3): *n* (%)	287 (37.0)
Sulcal widening grade (≥3): *n* (%)	511 (66.0)
Ventricle enlargement grade (≥3): *n* (%)	521 (67.2)
3MSE (score): mean (SD)	88.4 (9.3)
WAIS-IV coding subtest (score): mean (SD)	44.0 (15.8)
COWAT f,a.s test (score): mean (SD)	24.3 (11.5)
CVLT long delay free recall (score): mean (SD)	5.4 (2.3)

CVD, Cardiovascular Disease; CDCAI, Cerebrovascular Disease and its Consequences in American Indians; CIMT, Carotid Intima-Media Thickness; WMH, White Matter Hyperintensities; 3MSE, Modified Mini-Mental State Examination; WAIS, Wechsler Adult Intelligence Scale; COWAT, Controlled Oral Word Association Test; CVLT, California Verbal Learning Test.

Carotid atherosclerosis, as evidenced by plaque, was found in 57% of participants; most had plaque in one carotid arterial segment (55.5%). The overall mean of CIMT was 0.7 mm.

The mean age at the follow-up CDCAI study was 72.8 years. Brain MRI findings included infarcts noted in 33.3% and hemorrhages in 5.8% of participants. More than one-third (37.0%) of participants had abnormal WMH grade, 66.0% had abnormal sulcal widening, and 67.2% had abnormal ventricle enlargement.

The distributions of scores for each cognitive test were recently described (27). The 3MSE was heavily right-skewed, with a mean score of 88.4. The CVLT long delay free recall test was slightly right-skewed, with a mean score of 5.4. The WAIS coding subtest showed an approximately normal distribution based on visual inspection of the histogram, with a mean score of 44 (27). The COWAT was also normal with a mean score of 24.3. There are no established clinical cutoffs for these cognitive tests to indicate cognitive impairment validated in this population (27–29).

### 3.1 Relation of carotid atherosclerosis and brain MRI measures

Among the three carotid atherosclerosis measures, presence of plaque, plaque score, and CIMT were differentially associated with specific brain MRI features.

#### 3.1.1 Infarcts

No significant associations were observed between presence of plaque, plaque score, or CIMT and the odds of cerebral infarcts.

#### 3.1.2 Hemorrhages

Similarly, none of the carotid measures were significantly associated with the presence of cerebral hemorrhages.

#### 3.1.3 White matter hyperintensity (WMH)

Carotid measures were not significantly associated with WMH grade in this cohort.

#### 3.1.4 Sulcal widening

Greater CIMT was associated with more severe sulcal widening, after adjustment for all covariates. This association remained statistically significant in a sensitivity analysis restricted to participants who were free of CVD at the third examination (Model 5). No significant association was observed between sulcal grade and plaque presence or plaque score.

#### 3.1.5 Ventricular enlargement

Presence of plaque and plaque score were not significantly associated with ventricle grade. CIMT was also not significantly associated with ventricle enlargement.

### 3.2 Relation of carotid atherosclerosis and cognitive test scores

As shown in Table 2, presence of plaque and plaque score were significantly associated with worse COWAT score in all of the models adjusted for potential confounders. Additional adjustment in Model 6 for the MRI markers (infarct, hemorrhage, WMH grade, sulcal grade, and ventricle grade) did not alter the significant association between presence of plaque and worse COWAT score. Presence of plaque and plaque score were not associated with 3MSE score, WAIS score, or CVLT long delay free recall score.

**Table 2:** Odds ratios and beta coefficients with 95% confidence intervals and *P*-values from regression models of carotid atherosclerosis markers with MRI-defined structural brain indices and cognitive test scores (models 1–6).

Model 1: Adjusted for age, sex, study center									
	Presence of Plaque			Plaque Score			CIMT		
LOGISTIC	OR	CI	Pval	OR	CI	Pval	OR	CI	Pval
Infarct	1.17	0.84, 1.65	0.36	1.17	0.86, 1.60	0.31	1.16	0.31, 4.31	0.83
Hemorrhage	1.18	0.58, 2.39	0.65	1.17	0.62, 2.21	0.64	2.91	0.35, 24.03	0.32
POISSON	beta	CI	Pval	beta	CI	Pval	beta	CI	Pval
WMH grade	0.04	−0.03, 0.12	0.28	0.03	−0.04, 0.10	0.43	0.13	−0.15, 0.40	0.36
Sulcal grade	**0**.**06**	**0.01, 0.11**	**0**.**03**	**0**.**06**	**0.01, 0.11**	**0**.**01**	**0**.**23**	**0.03, 0.43**	**0**.**02**
Ventricle grade	**0**.**08**	**0.02, 0.14**	**0**.**01**	**0**.**06**	**0.00, 0.12**	**0**.**03**	0.12	−0.11, 0.35	0.30
LINEAR	beta	CI	Pval	beta	CI	Pval	beta	CI	Pval
3MSE	−0.81	−2.23, 0.61	0.26	−0.81	−2.11, 0.48	0.22	0.55	−5.22, 6.32	0.85
WAIS	−1.49	−3.69, 0.70	0.18	−1.44	−3.35, 0.48	0.14	3.01	−5.51, 11.54	0.49
COWAT	**−2**.**45**	**−4.19, −0.71**	**0**.**01**	**−2**.**30**	**−3.84, −0.75**	**0**.**00**	0.17	−6.80, 7.14	0.96
CVLT LD FR	−0.08	−0.43, 0.27	0.65	−0.10	−0.40, 0.21	0.54	−0.58	−1.93, 0.77	0.40
Model 2: Adjusted for covariates in Model 1 plus education, smoking, alcohol									
	Presence of Plaque			Plaque Score			CIMT		
LOGISTIC	OR	CI	Pval	OR	CI	Pval	OR	CI	Pval
Infarct	1.17	0.83, 1.65	0.36	1.18	0.86, 1.62	0.29	1.3	0.34, 4.92	0.70
Hemorrhage	1.2	0.60. 2.41	0.61	1.2	0.63, 2.28	0.58	2.87	0.33, 25.04	0.34
POISSON	beta	CI	Pval	beta	CI	Pval	beta	CI	Pval
WMH grade	0.04	−0.04, 0.11	0.36	0.02	−0.05, 0.10	0.52	0.11	−0.16, 0.39	0.42
Sulcal grade	0.05	0.00, 0.11	0.06	**0**.**05**	**0.01, 0.10**	**0**.**03**	**0**.**23**	**0.03, 0.43**	**0**.**02**
Ventricle grade	**0**.**08**	**0.02, 0.14**	**0**.**01**	**0**.**06**	**0.00, 0.12**	**0**.**04**	0.12	−0.11, 0.36	0.30
LINEAR	beta	CI	Pval	beta	CI	Pval	beta	CI	Pval
3MSE	−0.86	−2.15, 0.44	0.19	−1.00	−2.18, 0.18	0.10	−0.13	−5.31, 5.04	0.96
WAIS	−1.17	−3.10, 0.76	0.23	−1.28	−3.05, 0.49	0.16	1.98	−5.58, 9.54	0.61
COWAT	**−2**.**28**	**3.89, −0.65**	**0**.**01**	**−2**.**25**	**−3.74, −0.76**	**0**.**00**	−1.97	8.21, 4.27	0.54
CVLT LD FR	−0.10	−0.44, 0.25	0.58	−0.12	−0.42, 0.19	0.44	−0.67	−2.03, 0.70	0.34
Model 3: Adjusted for covariates in Model 2 plus BMI, fasting glucose, SBP, LDL cholesterol									
	Presence of Plaque			Plaque Score			CIMT		
LOGISTIC	OR	CI	Pval	OR	CI	Pval	OR	CI	Pval
Infarct	1.08	0.75, 1.54	0.68	1.09	0.79, 1.51	0.59	0.83	0.20, 3.37	0.79
Hemorrhage	1.24	0.58, 2.67	0.58	1.25	0.63, 2.46	0.52	3.85	0.34,43.61	0.28
POISSON	beta	CI	Pval	beta	CI	Pval	beta	CI	Pval
WMH grade	0.02	−0.06, 0.10	0.61	0.01	−0.07, 0.08	0.89	0	−0.29, 0.29	1.00
Sulcal grade	0.04	−0.02, 0.09	0.21	0.04	−0.01, 0.09	0.10	**0**.**23**	**0.02, 0.44**	**0**.**03**
Ventricle grade	0.06	0.00, 0.13	0.06	0.039	−0.02, 0.10	0.19	0.039	−0.20, 0.28	0.75
LINEAR	beta	CI	Pval	beta	CI	Pval	beta	CI	Pval
3MSE	−0.94	−2.30, 0.43	0.18	−1.07	−2.30, 0.16	0.09	0.32	−526, 5.89	0.91
WAIS	−0.64	−2.67, 1.39	0.54	−0.71	−2.59, 1.18	0.46	5.93	−2.01 13.87	0.14
COWAT	**−2**.**09**	**−3.74, −0.44**	**0**.**01**	**−2**.**01**	**−3.55, −0.47**	**0**.**01**	−0.49	−7.17, 6.19	0.89
CVLT LD FR	−0.07	−0.42, 0.28	0.70	−0.11	−0.41, 0.20	0.50	−0.69	−2.12, 0.74	0.35
Model 4: Adjusted for covariates in Model 3 plus time-between-exam									
	Presence of Plaque			Plaque Score			CIMT		
LOGISTIC	OR	CI	Pval	OR	CI	Pval	OR	CI	Pval
Infarct	1.06	0.74, 1.51	0.76	1.08	0.78, 1.49	0.66	0.85	0.21, 3.47	0.82
Hemorrhage	1.26	0.58, 2.73	0.56	1.6	0.64, 2.49	0.51	3.81	0.33, 43.59	0.28
POISSON	beta	CI	Pval	beta	CI	Pval	beta	CI	Pval
WMH grade	0.02	−0.06, 0.10	0.62	0	−0.07, 0.08	0.90	0	−0.29, 0.29	1.00
Sulcal grade	0.037	−0.02, 0.09	0.19	0.04	−0.01, 0.09	0.09	**0**.**23**	**0.02, 0.44**	**0**.**03**
Ventricle grade	0.046	−0.02, 0.11	0.15	0.03	−0.03, 0.08	0.36	0.06	−0.18, 0.29	0.64
LINEAR	beta	CI	Pval	beta	CI	Pval	beta	CI	Pval
3MSE	−0.77	−2.14, 0.61	0.27	−0.93	−2.16, 0.31	0.14	0.24	−5.37, 5.85	0.93
WAIS	−0.41	−2.44, 1.62	0.69	−0.51	−2.40. 1.38	0.60	5.59	−2.27, 13.46	0.16
COWAT	**−2**.**03**	**−3.69, −0.36**	**0**.**02**	**−1**.**96**	**3.51, −0.41**	**0**.**01**	−0.59	−7.28, 6.09	0.86
CVLT LD FR	−0.02	−0.37, 0.34	0.92	−0.06	−0.37, 0.25	0.70	−0.74	−2.16, 0.68	0.31
**Model 5**: Adjusted for covariates in Model 4 plus includes participants CVD-free at baseline									
	Presence of Plaque			Plaque Score			CIMT		
LOGISTIC	OR	CI	Pval	OR	CI	Pval	OR	CI	Pval
Infarct	1	0.67, 1.41	0.90	1.3	0.71, 1.48	0.89	0.54	0.12, 231	0.41
Hemorrhage	1.18	0.53, 2.59	0.69	1.31	0.57, 2.97	0.52	2.9	0.21, 41.43	0.43
POISSON	beta	CI	Pval	beta	CI	Pval	beta	CI	Pval
WMH grade	0.026	−0.06, 0.11	0.54	0.03	−0.05, 0.11	0.47	0	−0.31, 0.30	0.97
Sulcal grade	0.03	−0.03, 0.09	0.29	0.04	−0.01, 0.10	0.12	**0**.**23**	**0.01, 0.45**	**0**.**05**
Ventricle grade	0.04	−0.02, 0.11	0.22	0.035	−0.03, 0.10	0.26	0.06	−0.19, 0.31	0.63
LINEAR	beta	CI	Pval	beta	CI	Pval	beta	CI	Pval
3MSE	−0.62	−2.04, 0.81	0.39	−0.59	−1.97, 0.78	0.40	0.43	−5.49, 6.36	0.89
WAIS	0.34	−1.73, 2.41	0.75	0.58	−1.40, 2.55	0.57	6.06	−1.98, 14.09	0.14
COWAT	**−1**.**96**	**−3.66, −0.25**	**0**.**02**	**−1**.**89**	**−3.52, −0.26**	**0**.**02**	−1.32	−8.19, 5.55	0.71
CVLT LD FR	0.03	−0.33, 0.39	0.86	0.01	−0.34, 0.36	0.96	−0.55	−2.06, 0.95	0.47
Model 6: Adjusted for covariates in Model 5 plus MRI markers									
	Presence of Plaque			Plaque Score			CIMT		
LINEAR	beta	CI	Pval	beta	CI	Pval	beta	CI	Pval
MSE	−0.6	−1.9, 0.8	0.421	−0.7	−2.0, 0.6	0.275	1.6	−3.5, 6.7	0.539
WAIS	0.2	−1.8, 2.2	0.869	0	−1.9, 1.9	0.997	5.9	−1.5, 13.4	0.119
COWAT	**−1**.**9**	**−3.5, −0.2**	**0**.**032**	**−1**.**8**	**−3.4, −0.2**	**0**.**026**	−0.3	−7.0, 6.3	0.923
CVLT LD FR	0.04	−0.3, 0.4	0.815	−0.04	−0.4, 0.3	0.793	−0.6	−2.0, 0.8	0.424

CVD, Cardiovascular Disease; CIMT, Carotid Intima-Media Thickness; WMH, White Matter Hyperintensities; 3MSE, Modified Mini-Mental State Examination; WAIS, Wechsler Adult Intelligence Scale; COWAT, Controlled Oral Word Association Test; CVLT LD FR, California Verbal Learning Test–Long Delay Free Recall.

Bold values indicate statistical significance at *p* ≤ 0.05.

CIMT was not associated with any of the cognitive performance tests.

## 4 Discussion

This study identified two key findings related to markers of carotid atherosclerosis, including CIMT, presence of carotid plaque, and plaque burden, with important implications for aging brain health in American Indian populations. First, increased CIMT was significantly and robustly associated with sulcal widening, a hallmark of neurodegeneration, even after comprehensive covariate adjustment. Second, presence of carotid plaque and higher plaque burden were independently associated with poorer verbal fluency performance on the COWAT, a finding that remained after controlling for the same set of risk factors. These associations, observed over a 13-year follow-up period, suggest that subclinical atherosclerosis in midlife may be associated with later-life neurodegenerative brain changes and lower cognitive performance in this underserved and high-risk population.

The association between increased CIMT and sulcal widening, a marker of neurodegeneration and atrophy, aligns with findings reported in previous studies (30, 31); however, to our knowledge, ours is the first study to examine this relationship in a large, population-based study of American Indians.

The increased CIMT may reflect an underlying cerebral vasculopathy involving both large-vessel remodeling and dysfunction in intracranial small vessels, leading to compromised cerebral microcirculation. This vascular pathology can reduce cerebral perfusion (32) and oxygen delivery (33), contributing over time to neurodegenerative changes and structural brain injury (34, 35). In addition, CIMT has been shown to correlate independently with arterial stiffness (36), a well-established vascular marker associated with a greater burden of structural brain damage (37). However, the directionality and temporal sequence between intima-media thickening and arterial stiffening remain areas of ongoing investigation (38). As both processes may contribute to neurodegeneration through chronic cerebral hypoperfusion and vascular insufficiency, future studies comparing their respective and combined roles could enhance understanding of vascular pathways leading to brain atrophy and cognitive decline.

Prior studies have reported inconsistent findings regarding associations between carotid atherosclerosis and MRI-defined features of vascular brain injury. Several studies have identified associations between carotid atherosclerosis and white matter hyperintensities (WMH) or infarcts (39, 40), yet others have not (31, 41). In our cohort, we did not observe significant associations between carotid atherosclerosis and the presence of infarcts or WMH. One possible explanation is that carotid atherosclerosis may primarily affect cortical structures through the mechanisms described above, which do not necessarily result in WMH. In contrast, WMH often arise from arteriolosclerotic changes associated with chronic hypertension, aging, or cerebral amyloid angiopathy (42). Supporting this distinction, prior work has demonstrated that neurodegenerative brain changes can occur independently of WMH (43), and that WMH burden explains only a modest portion of variability in brain atrophy (44). Additionally, cortical thinning has been observed in individuals with asymptomatic carotid stenosis, even in the absence of infarcts (45), suggesting a direct impact of carotid disease on cortical integrity that is independent of small vessel disease.

In contrast to the consistent association observed between increased CIMT and sulcal widening, we did not find similarly robust associations for the other two carotid atherosclerosis markers, plaque presence and plaque score. While both were initially associated with higher sulcal and ventricular grades in models adjusted for sociodemographic and behavioral factors, these associations were attenuated and no longer significant after adjusting for clinical CVD risk factors. This pattern suggests that the observed associations may have been confounded by coexisting vascular risk factors, and that sulcal widening, rather than ventricular enlargement, is the structural MRI marker of neurodegeneration most consistently associated with carotid atherosclerosis in our sample. It's worth noting that our regression models were adjusted for carefully selected and accurately measured covariates *a priori*. However, the high prevalence and overlapping causal influence of key sociodemographic, behavioral, and CVD risk factors in this population may have contributed to residual confounding. These interrelated factors could have attenuated the observed associations between these two markers of carotid atherosclerosis and the markers of neurodegeneration and atrophy even after thorough adjustment. Covariates such as age, hypertension, diabetes, and smoking have been shown to be associated with WMH, ventricular enlargement, and sulcal widening (8, 18). Residual confounding is a common limitation in observational studies, even when confounders are well measured and appropriately modeled. To help address this challenge, future studies could incorporate repeated measurements of vascular risk factors and brain MRI markers over time. Such data would allow better modeling of long-term exposures and temporal relationships between vascular and brain changes, which may help clarify causal pathways.

We observed a consistent and significant association between the presence of plaque in the carotid artery and worse COWAT score. A similar finding was observed in the Framingham study, where researchers found that ≥50% internal carotid stenosis was associated with poorer performance on executive function assessing three domains of cognitive function, namely, verbal memory, executive function and nonverbal memory function (1). Our finding of a strong association between plaque score and poorer performance on COWAT is consistent with previous research. In the Rotterdam Study, a population-based prospective cohort study among 7,983 elderly participants, plaque score was associated with an increased risk of dementia and mortality (46). The Survival and Outcome After Stroke Study showed that both the number of carotid plaques and the number of carotid arteries with plaque were significantly associated with cognitive impairment after adjusting potential confounders (47). Taken together, our findings and those from prior studies suggest that structural atherosclerotic changes in the carotid arteries, even in the absence of clinical stroke, may adversely impact cognitive performance, particularly in domains dependent on frontal lobe function such as verbal fluency.

Several underlying mechanisms may explain the association between carotid atherosclerosis and poorer cognitive performance on the COWAT. Carotid atherosclerosis has been linked to cognitive impairment with or without brain infarction, and previous research has pointed to embolization and hypoperfusion as possible pathways (48). Carotid atherosclerosis has been linked to cerebral hypoperfusion, which may lead to brain atrophy, dementia, and other cognitive deficits, according to research (49). We found that carotid atherosclerosis was significantly associated with atrophic changes in the brain (in the form of sulcal widening) and cognitive impairment (in the form of deficits in verbal fluency and executive function). This raises the possibility that the link between carotid atherosclerosis and cognitive impairment is mediated, at least in part, by degenerative changes manifested by sulcal widening. These findings were consistent with previous studies that have shown that worse sulcal widening was related to executive dysfunction (50) and a deficit in verbal fluency (51). Although language use was assessed in CDCAI and SHS Phase VII, bilingualism and number of spoken languages were not formally measured, limiting our ability to account for their potential influence on verbal fluency performance.

In contrast to the significant associations observed for plaque presence and plaque burden, CIMT was not related to poorer cognitive performance in our study. While increased CIMT has been associated with losses in verbal and nonverbal memory function in some prior studies, for example, the Framingham Offspring Study of 1,975 individuals without stroke or dementia (41), we did not observe such associations. One possible explanation is that CIMT was measured approximately 13 years prior to cognitive testing and may therefore have underestimated the extent of subclinical vascular disease present at the time cognition was assessed.

We also evaluated the effect of MRI markers on the association between carotid atherosclerosis and cognitive performance. The associations remained significant after adjusting for the MRI measures. This finding study suggests that carotid atherosclerosis could also be an independent marker of poor cognitive performance.

Our study has several notable strengths. The SHS is a large population-based cohort study with long-term follow-up. The assessment of carotid measures performed approximately 13 years prior to the assessments of brain MRI and cognitive testing measures provides stronger evidence of relationships between carotid atherosclerosis measures, brain MRI markers, and cognitive performance than cross-sectional analyses. Multiple traditional CVD risk factors were included in the adjusted models to minimize residual confounding. However, our analysis also has limitations. As with any cohort study of older adults, loss to follow-up due to mortality and non-participation due to frailty was unavoidable; hence, confounding due to survival bias is possible. However, previous analyses of the extent or degree of selective survival from the SHS baseline to CDCAI examinations have not identified evidence of such bias (9). The cognitive assessments used in this study have been psychometrically evaluated in older American Indian adults from the Strong Heart Study. The 3MSE showed strong internal consistency and a unidimensional factor structure, with measurement invariance by sex and age, although scores were influenced by education, depression, and bilingualism (29). The MoCA demonstrated good convergent validity with the 3MSE and showed unifactorial validity (52). COWAT scores were similarly reliable and valid but varied by educational attainment and language use (53). Although these cognitive screening tools performed well at the group level, their ability to accurately identify dementia was weaker than in other populations, likely due to lower formal education and cultural-linguistic differences. As such, they are appropriate for population-level studies but should be interpreted cautiously in clinical settings (10). In addition, since brain MRI indices and measures of cognitive performance were not assessed at baseline, the temporal sequence between exposure and outcome could not be ascertained. However, it is unlikely that reverse temporality would explain any observable associations.

In conclusion, within a community-based sample of American Indian adults, the presence of certain markers of carotid atherosclerosis in mid-life was associated, at an older age, with sulcal widening, a structural MRI marker of vascular and neurodegenerative brain aging, and poorer cognitive performance. Notably, carotid atherosclerosis was associated with aspects of cognition independent of MRI-defined structural brain changes. To our knowledge, this is the first to report these associations in this severely understudied population with a high prevalence of CVD risk factors. These findings suggest that carotid atherosclerosis may be a distinct risk factor for lower cognitive performance in elderly American Indians.

Diagnosing and treating carotid atherosclerosis at an early stage, along with interventions to control or modify CVD risk factors, may be a potential strategy for the prevention of accelerated brain atrophy or cognitive aging, potentially leading to healthier older American Indians. Further longitudinal studies with repeated measures of both atherosclerosis and cognitive impairment are needed, as well as determinants of how early treatment of atherosclerosis may prevent cognitive changes.

## Data Availability

The data analyzed in this study is subject to the following licenses/restrictions: the data were collected, analyzed, and reported under agreements made with the sovereign tribal nations that have partnered in this research, which preclude routine modes of data sharing. Requests to access the dataset from qualified researchers trained in human subject confidentiality protocols may be sent to the Strong Heart Study Coordinating Center at https://strongheartstudy.org/. Requests will be reviewed by tribal research partners before data may be released. This policy is consistent with the “NIH Policy for Data Management and Sharing: Responsible Management and Sharing of American Indian/Alaska Native Participant Data. Requests to access these datasets should be directed to www.strongheartstudy.org.
